# Baseline CD4+ T lymphocyte cell counts, hepatitis B and C viruses seropositivity in adults with Human Immunodeficiency Virus infection at a tertiary hospital in Nigeria

**DOI:** 10.4314/pamj.v9i1.71178

**Published:** 2011-05-21

**Authors:** Ajayi Ebenezer Adekunle, Ajayi Akande Oladimeji, Adegun Patrick Temi, Ajayi Iyiade Adeseye, Ojo Abiodun Akinyeye, Raimi Hussean Taiwo

**Affiliations:** 1 Department of Medicine, University Teaching Hospital, Ado Ekiti, Nigeria; 2 Department of Surgery, University Teaching Hospital, Ado Ekiti, Nigeria; 3 Department of Ophthalmology, University Teaching Hospital, Ado Ekiti, Nigeria; 4 Department of Hematology, University Teaching Hospital, Ado Ekiti, Nigeria

**Keywords:** HIV AIDS infection, CD4+ T-lymphocyte cell counts, Hepatitis B virus infection

## Abstract

**Background:**

Ekiti State of Nigeria is known to have the lowest prevalence of HIV in Nigeria. University Teaching Hospital (UTH), Ado Ekiti was recently upgraded to serve as one of the three centres for HIV/AIDS referral, diagnosis and treatment in Ekiti State. We evaluated the baseline immunologic and biochemical parameters of patients presenting at the ART clinic of University Teaching Hospital, Ado Ekiti, Ekiti State.

**Methods:**

All HIV seropositive patients not yet on antiretroviral therapy, who presented at the ART Clinic within the study period had at the first visit to the clinic, their blood sample taken for CD4 cell counts estimation, HBsAg and anti- HCV screening, ALT, AST as well as hemoglobin estimation as part of the routine workup to assess their disease health status and need for antiretroviral therapy. Statistical significance was taken as p< 0.05.

**Results:**

A total of 273 patients comprising 79 (28.9%) males and 194 (71.1%) females were included in the study (F:M = 2.46: 1). The mean age of the study population was 36.21± 10.20 years with mean age of males (39.52 ± 9.95years) significantly higher than females (34.88 ± 10.02; p=0.001). The overall prevalence of HBsAg in the study population was 6.6% with a sex specific prevalence of 8.1% and 6% for males and females, respectively. No statistically significance difference in the mean serum alanine transaminase, serum aspartate transaminase, hemoglobin and CD4+ T- Lymphocytes cell count of those who had HBsAg negative status compared to those who had HBsAg positive status. Two (0.7%) of the patients had positive serum anti HCV antibodies. The CD4+ T- Lymphocytes cell count ranged between 5 – 1050 cells/µl with a mean of 286.19 ± 233.31 cells/µl. The majority of patients (71.8%) had a CD4+ T- Lymphocytes cell count < 350 cells/µl.

**Conclusion:**

At the time of presentation, majority of our patients had a CD4+ T- Lymphocytes cell count less than 350 cells/µl consistent with significant immune-suppression. More sustained and vigorous awareness campaigns still need to be done in Ekiti State to diagnose this disease early. There is also a need to accelerate the integration of hepatitis B virus screening and treatment programme into HIV/AIDS programme because of the morbidity and mortality implication of HBV and HIV co-infection.

## Background

HIV infection is a global pandemic. By the end of 2007 it was estimated that about 33.2 million people were living with HIV in the world with more than 60% of the infected population in sub-Saharan Africa [1]. In Nigeria, the prevalence of HIV among adults during the year 2007 was 3.1% [2]. In that year 2007, 170,000 deaths of the estimated 2.6 million people living with HIV/AIDS were reported. In response to the global efforts at improving care and treatment, the Nigeria Government in collaboration with various partners run HIV care and treatment that included the provision of free antiretroviral drugs and drugs for opportunistic infections.

Despite the enormous attention being paid to early diagnosis and treatment of HIV/AIDS worldwide, reports still showed that most patients still present late for care [3–5]. The impact of this on morbidity and mortality vis-à-vis the reduced immunologic status at presentation had also been documented [6, 7]. In addition, the common routes of infection shared by HIV, HBV and HCV [8] have generated interests in co-infection between HIV, HBV and/or HCV. As a matter of facts, about 5% to 10% of HIV patients harbor persistent serum HBsAg and therefore suffer from chronic hepatitis B [9]. Progression to end-stage liver disease occurs more quickly in HIV/HBV-coinfected patients; this is characteristic in the absence of significant elevations in liver enzymes, as inflammatory phenomena in the liver are ameliorated in HIV infection although paradoxically fibrogenesis is enhanced. Liver disease is currently one of the leading causes of morbidity and mortality in HIV – infected individuals with chronic hepatitis B and hepatitis C being the major causes of hepatic disease in this population [10]. Though, screening for hepatitis B and C viruses in HIV-infected individuals is becoming widespread, integration of the treatment of these viral hepatitides has not been achieved in most countries, including Nigeria.

The objectives of this study were to: (1) determine the baseline CD4+ T Lymphocytes cell count and haemoglobin level in antiretroviral naïve HIV patients; (2) determine the prevalence rates at baseline of HBsAg and anti- hepatitis C antibody (HCV-ab) sero-positive status in this population of HIV patients who presented at the ART Clinic of a recently upgraded centre for HIV/AIDS referral, diagnosis and treatment in Ekiti State, southwestern Nigeria (where HIV prevalence at 1% is the lowest in Nigeria).

## Methods

This study was carried out at the medical department of the University Teaching Hospital (UTH), Ado-Ekiti, Nigeria, in the period January 2009-March 2010 (15 months period). This centre is one of the three recently upgraded centres in Ekiti State, Southwestern Nigeria to provide care and treatment including provision of free antiretroviral drugs for HIV/AIDS referral, diagnosis and treatment. Usually, in our Centre, HIV Testing and Counseling (HCT) is routine in all the clinical service areas, in addition to the quarterly community HCT outreaches where subjects’ capillary blood samples are screened for HIV by the use of two nationally approved rapid HIV kits run in parallel (Determine^TM^ HIV-1/2, Abbot Laboratories, USA and STAT-PAK^R^ HIV- 1/2, Chembio Diagnostic Systems, Inc, USA). Those who are HIV positive are thereafter referred to ART clinic for enrollment into HIV care. A total of 7,122 subjects were thus screened during the study period.

All HIV seropositive patients who were antiretroviral therapy naïve and who presented at the ART Clinic within the study period had at the first visit to the clinic, their blood sample taken for CD4 cell counts estimation, HBsAg and anti- HCV screening, ALT, AST as well as hemoglobin estimation as part of the routine workup to assess their disease health status and need for antiretroviral therapy. Freshly taken blood sample drawn into a 2-mL Ethylene Diamine Tetra-acetic Acid (EDTA) anticoagulant bottle and processed within 2 hours of venesection, was used to estimate CD4+ T Lymphocytes cell counts; screened for HBsAg and HCV-ab; as well as estimation of haemoglobin concentration. Samples for CD4+ T Lymphocytes cell counts were prepared and run on the Cyflow SL 3 (Partec Inc., Germany) according to the manufacturer′s instructions. Qualitative detection of HBsAg and HCV-ab in plasma was done using rapid diaspot one step strip (Sam Tech Diagnostics). Haemoglobin concentration estimation was done with the use of Haemocue haemoglobin concentration machine (HaemoCue AB Angelholm, Sweden). Alanine transaminase (ALT) and aspartate transaminase (AST) were determined by colorimetric method using the Randox kit (Randox Laboratories Ltd, UK) with absorbance read on a spectrophotometer at a wavelength range of 530-560 nanometer.

The laboratory dedicated to HIV care in our Centre is routinely subjected to internal quality control assessment using the Manufacturers’ prepared kit or test solution as well as quarterly external quality control using a reference laboratory supervised by the Institute of Human Virology of Nigeria.

The protocol for this study was approved by the Research and Ethic Committee of the University Teaching Hospital, Ado Ekiti.

### Statistical analysis

Data were expressed as mean ± standard deviation (SD), and frequency and were expressed as numbers and percentages. Computation of P values was done by t-test and chi-squared analysis. P < 0.05 was considered statistically significant. All statistical analyses were performed with the commercially available computer program SPSS 11.0 (SPSS Inc., Chicago, IL).

## Results

The mean age of the 273 patients was 36.21± 10.20 years (range 16 -79 years). There were 79 (28.9%) males and 194 (71.1%) females giving a male to female ratio of 1: 2.46. The males, with a mean age of 39.52 ± 9.95years were significantly older than females whose mean age was 34.88 ± 10.02; p=0.001. Majority of the patients (71.1%) were within the age group 21 – 40 years. The majority (79.2%) was married at the time of presentation and only 8% of them did not have any form of formal education.

Eighteen patients were HBsAg sero-positive giving a sero-positive prevalence of 6.6% among the study population. Frequency of positive serum HBsAg was 8.1% and 6% among males and females respectively. When the HBsAg sero-negative patients were compared with those who were HBsAg sero-positive, there was no significant difference in their mean serum ALT (20.84± 18.26 vs. 13.39± 11.20), AST (19.43 ± 15.17 vs. 12.14± 5.49), hemoglobin (10.23± 2.33 vs. 11.46± 1.28) and CD4+ T Lymphocytes cell count (258.13± 211.16 vs. 234.07± 178.34). Serum anti- HCV antibody was positive in 2(0.7%) of the 273 patients studied. Distribution of the number of seropositive patients for HBsAg and anti-HCV antibody according to age groups is shown in Table 1 while Table 2 provides the baseline ALT, AST and Hemoglobin in different strata of CD4+ T lymphocyte cell count.

**Table 1:** Baseline distribution of CD4- T lymphocyte cell count, Hemoglobin, and HBsAg and HCV positive status by age groups in a group of adults with Human Immunodeficiency Virus infection at a tertiary hospital in Nigeria

	≤20	21-30	31-40	41-50	51-60	>60	P value
	
Variable	(n=7)	(n=91)	(n=103)	(n=34)	(n=8)	(n=3)
CD4 cell count (cells/µl)	492.33±355.15	296.49±225.41	285.88±243.18	248.62±219.20	263.22±182.99	254.00±81.62	0.294
Hemoglobin(mg/dl)	11.00±3.16	10.23±2.54	10.40±2.27	10.48±1.73	9.47±2.36	10.92±1.29	0.783
HBsAg positive status(n)	2	8	6	0	2	0	0.682
HCV positive status (n)	0	1	0	1	0	0	0.234

**Table 2:** Baseline ALT, AST and Hemoglobin in different strata of CD4- T lymphocyte cell count in a group of adults with Human Immunodeficiency Virus infection at a tertiary hospital in Nigeria

	Strata of CD4- T lymphocyte cell count				
	
Variable	≥ 500	350-499	200-349	<200	P value
Serum aspartate transaminase(mmol/l)	15.00±5.70	12.00±6.71	20.72±19.72	19.35±15.00	0.35
Serum alanine transaminase(mmol/l)	20.40±12.86	15.86±7.61	20.83±18.47	17.43±14.08	0.59
Haemoglobin(mg/dl)	10.97±2.37	10.87±1.89	10.83±1.93	9.81±2.36	0.01

The mean CD4+ T Lymphocytes cell count of the study population was 286.19 ± 233.31 (range 5- 1,051). The mean CD4+ T Lymphocytes cell count of the males was 293.95±244.53, while that for females was 283.47 ± 229.96; p= 0.77. The CD4+ T Lymphocytes cell count of majority of the patients (71.8%) was < 350 cells/µl.

The mean haemoglobin was 10.34 ± 2.25 g/dl. Males (11.06 ±2.18g/dl) had significantly higher mean haemoglobin than females (10.05 ± 2.22g/dl;p=0.004). Sixty five percent had hemoglobin ≥ 10g/dl. Among the males, 22.8% had hemoglobin < 10g/dl while 39.9% of the females had haemoglobin < 10g/dl (p= 0.016). Mean hemoglobin reduced progressively as the serum CD4+ T Lymphocytes cell count declined.

## Discussion

In this study, majority of the patients first presented to the centre at a time when the CD4+ T Lymphocytes cell count was already in the range requiring commencement of ART. This shows that most of our patients still present late to the hospital. Quite a large percentage (45.5%) of our patients had a CD4+ T Lymphocytes cell count < 200 cells/ µL at presentation and about 72% had CD4+ T Lymphocytes cell count < 350 cells/ µL. Earlier reports from Northern Nigeria showed that 50-54% of HIV patients had CD4+ T Lymphocytes cell count < 200 cells/ µL at presentation [11,12]. Other African ART programs have also reported low CD4+ T Lymphocytes cell count at enrollment for adult ART [13,15]. In a study in India [16] where 43 HIV patients who were antiretroviral naïve were studied to determine correlation between CD4+ T Lymphocytes cell count and plasma viral load, all the patients had their CD4+ T Lymphocytes cell count < 200 cells/ µL at presentation. The major reason for this high proportion of HIV patients presenting with low CD4+ T Lymphocytes cell count is late presentation.

Late presentation of HIV patients for care is definitely not a problem that is peculiar to our environment. Between 10% to 30% of HIV-infected individuals in the Western world reported to present late for care [3,4] and higher percentage in developing countries, particularly in sub-Saharan Africa, South-East Asia and South America, because of the limited access to healthcare and HIV treatment [5]. Stigma and delay in seeking health care, reluctance to utilizing voluntary testing and counseling services, and health system delays in referral and ART initiation are other possible explanations. Thus, priority must be given to identifying HIV-infected individuals and starting treatment earlier in the course of their illness, before they develop severe opportunistic infections. More sustained and vigorous awareness campaigns still need to be done to diagnose this disease early.

The mean hemoglobin in the population was low with males having significantly higher mean hemoglobin compared to females. Mean hemoglobin reduced progressively as the serum CD4+ T Lymphocytes cell count declined. It is known that the incidence of anemia increases with progression of HIV infection [17] which is the case in the present study. Furthermore, in this population of HIV patients with low CD4+ T Lymphocytes cell count, anemia can be a feature of certain opportunistic diseases, like disseminated mycobacterial infection and parvovirus B19. Other etiologic factors that may be involved in the development of HIV-associated anemia include micronutrient deficiencies, immunological myelosuppression, impaired erythropoietin production and blood loss from intestinal opportunistic disease [18].

There is need for enhanced interest in HIV/HBV co-infection. Around 5% to 10% of HIV patients harbor persistent serum HBsAg and therefore suffer from chronic hepatitis B [9]. Progression to end-stage liver disease occurs more quickly in HIV/HBV-coinfected patients; this is characteristic in the absence of significant elevations in liver enzymes, as inflammatory phenomena in the liver are ameliorated in HIV infection although paradoxically fibrogenesis is enhanced. The overall prevalence of HBsAg in our study population was 6.6% with a sex specific prevalence of 8.1% and 6% for males and females, respectively. However, the rate for HCV-Ab (0.7%) was lower than the rate for HBsAg. Liver enzymes in this study were found not to be significantly elevated. The rates of HBsAg and HCV-Ab co-infections with HIV in our study are comparable with findings in other studies. For instance, results of an earlier study in Nigeria conducted on 1779 HIV-positive patients revealed that the rates for HBsAg, HCV, and HBV/HCV coinfections were 11.9, 4.8, and 1%, respectively [19]. Moreover, in India, a study showed that the prevalence rate of HBsAg in HIV-positive patients was 3.4% while the rate for HCV-Ab was reported to be 0% [20]. The prevalence rates of co-infection with HBV and HCV in HIV patients have been variable worldwide depending on the geographic regions, risk groups and the type of exposure involved which may be different not only from country to country, but also in different regions of the same country [9,21]. It is known that sexual transmission of HCV is lower in comparison to HBV and it is transmitted mostly via injection (especially in drug addiction). This may explain why the rate of HIV/HCV in our study was low since sexual transmission is the commonest root of infection in our environment [8]. Undoubtedly, it is important to fully integrate HBV and HCV diagnosis in HIV-positive patients in order to take care of them and allot resources in health plans.

Interestingly, hepatitis B virus infection is preventable. This can be achieved by similar measures taken to prevent the transmission of HIV infection such as safe sex practice, safe handling of sharps, and avoidance of sharing of intravenous drug paraphernalia, among others. In addition, safe and effective vaccines with probably lifelong immunity against all hepatitis B virus serotypes and genotypes are currently available [22].

The majority of our study population was within the age group 21-40 years which is in keeping with our country National survey [23] and global findings on the age group of patients most affected with HIV [1]. We also found in the present study that more HIV- infected females (71.1%) presented for care at a significantly younger age (34.88 ± 10.02 years) compared with males (28.9%) with the mean age at presentation of 39.52 ± 9.95 years. This is similar to the findings by Otegbayo [19] et al and Ajayi et al [15]. However, this is in contrast with the National sero-prevalence survey reported in 2003 where there was higher ratio of males to females usually seen in early phase of epidemic but tend to reverse as the epidemic matures. Whether this reversal in ratio of males to females seen in maturing epidemic was the case in this study or evidence that HIV sero-positive women seek health care more than HIV sero-negative men are issues that future survey should elucidate. It must be noted, however, that women of reproductive ages may have better access to HIV testing and counseling (HCT) than men through antenatal care services where HCT is now made compulsory.

## Conclusion

At the time of presentation, majority of our patients had a CD4+ T Lymphocytes cell count less than 350 cells/µl consistent with significant immune-suppression. Despite the low prevalence of HIV in Ekiti State, more sustained and vigorous awareness campaigns still need to be done in order to diagnose this disease early and encourage early presentation at the health care facility for those who are sero-positive. There is also a need to accelerate the integration of hepatitis B virus screening and treatment programme into HIV/AIDS programme because of the morbidity and mortality implication of HBV and HIV co-infection.
